# Professional Freedom

**Published:** 1907-10-19

**Authors:** 


### PROFESSIONAL FREEDOM.

As we go to press we have received a long letter from the Secretary of the National Anti-Vivisection Hospital, which arrived too late to be dealt with this week.

